# Influence of nutrition and food on sleep—is there evidence?

**DOI:** 10.1007/s11325-023-02921-1

**Published:** 2023-09-23

**Authors:** Nikolaus C. Netzer, Kingman P. Strohl, Stephan Pramsohler

**Affiliations:** 1 Hermann Buhl Institute for Hypoxia and Sleep Medicine Research, University Innsbruck, Innsbruck, Austria; 2 Institute for Mountain Emergency Medicine, Terra X Cube, EURAC Research, Via Hypathia 2, 39100 Bozen, Italy; 3 Div. of Sport Medicine, Dept. of Medicine, University Hospitals Ulm, Ulm, Germany; 4 Div. Pulmonary Medicine, Dept. Internal Medicine, University Hospitals, Case Western Reserve University, Cleveland, OH USA

**Keywords:** Sleep, Nutrition, Food, Sleep quality, Adipocyte signaling, Melatonin

## Abstract

**Background:**

The influence of sleep disorders on metabolism, especially concerning obesity and diabetes, as well as obesity and obstructive sleep apnea, has been widely investigated. However, the effect of nutrition and the intake of certain foods on sleep has only recently gained attention. In recent years, there have been publications on intake of certain foods and certain diets regarding their influence on sleep, as well as activity of adipocytes and their effect on production of sleep hormones.

**Methods:**

Following PRISMA guidelines, we performed a PubMed search using the key words “sleep,” “sleep disorders,” “nutrition,” “food,” and “food intake” published from 2012 to 2022. We excluded by consensus all articles with diets and exercise programs or bariatric surgery for weight loss to treat sleep apnea, all articles on connections between sleep disorders and metabolic disorders, and articles concerning the influence of drugs on neuroactive substances.

**Results:**

Of the 4155 publications revealed, 988 had nutrition, metabolism, and sleep as the primary topic of research. Of these 988 publications, only 26 fulfilled the content requirements concerning the influence of certain food and diets on sleep or sleep disorders, including the influence of the gastrointestinal system and adipocytes on sleep hormones. None of the investigations revealed clear evidence of an effect of a certain diet or food on sleep. Epidemiologic surveys suggest that shortened or fragmented sleep and chronotype in adults influence nutrition and fat metabolism. Additionally, there is evidence that adipocyte signaling influences neuronal mediators and hormones of the sleep-wake cycle.

**Conclusion:**

There is no evidence of a direct influence of certain nutrition or food intake on sleep. Obesity via adipocyte signaling may influence the sleep-wake cycle, though the molecular research on this topic is based on animal studies.

## Introduction

The comorbidity of obesity and obstructive sleep apnea (OSA) has been a topic of sleep medicine research for the past 50 years. This research was stimulated by the observations on patients with Pickwickian syndrome [1–3]. It was clear that nutrition leading to obesity can cause sleep-disordered breathing. Likewise, reducing obesity with calorie-restricted diets may reduce severity of sleep apnea and decrease its symptoms [4].

After the first description of OSA by Jung and Kuhlo [5] and its classification by Guilleminault et al. [6], it was clear that diabetes mellitus and OSA [7, 8] were associated. Studies showed that treatment of OSA with CPAP improved diabetes and blood sugar levels but it was never shown that a sugar-free diet could prevent the development of OSA.

Early research investigated the influence of caffeine and other methylxanthines on sleep structure, sleep quality, and insomnia [9, 10]. This topic has been in vogue recently due to the increasing consumption of taurine- and caffeine-containing energy drinks in teenagers and adolescents. Energy drink consumption is thought to be a reason for fragmented and short sleep in school children and students [11, 12]. Caffeine is clearly a neuroactive substance but it is not regarded as a food substance.

Alcohol and its effects on the brain have been intensely studied in humans and animals. Alcohol is initially tiring and induces slow wave sleep when ethanol-enhanced gamma amino butyric acid (GABA) binds with its receptors [13]. As GABA disconnects from receptors and activates and deactivates monoamines, GABA has various effects on the sleep-wake cycle [14]. The effects of alcohol on the brain are similar for men and women [15].

There are substantial effects of highly neuroactive substances in drinks and medications such as marijuana, hops, and magic mushrooms on sleep and wakefulness. However, the effect of other foods and the effect of the amount of food remain on sleep are unclear. A traditional saying is: “Have breakfast like an emperor, eat lunch like a king, and dine like a beggar,” meaning a large breakfast gives you strength for the day’s work, a sufficient lunch gives you energy through the afternoon but does not tire you, and a light dinner lets you sleep well. The scientific evidence for this traditional life advice should be examined. Therefore, this review aimed to investigate the evidence that certain diets or amounts of food influence sleep and wakefulness.

## Methods

Following PRISMA guidelines, we performed a PubMed search with the key words “sleep,” “sleep disorders,” “nutrition,” and “food intake” published from 2012 to 2022. Studies that did not meet the content requirements regarding the influence of a particular food, diets, or timing of eating on sleep, or the sleep-wake cycle were excluded. We also excluded studies on the direct influence of alcohol or caffeine-like substances in addition to diets for weight loss to treat OSA.

## Results

A total of 4155 publications were initially found, but only 988 had nutrition, metabolism, and sleep as the primary topic of research. Of these 988 publications, 26 met the content requirements concerning the influence of certain foods and diets on sleep or sleep disorders. We discuss the content of these 26 studies (see Table 1) in this review.

**Table 1:** List of reviewed articles

Author Year	Study type	Study objective	Instruments used	*N*	Outcome
Machado et al. (2022)	Review	White and brown adipose tissue	Literature search	*n* = 357 articles	AT is influenced by multiple factors including circadian rhythm
Agil et al. (2021)	RCT	Effect of melatonin on BAT	Mitochondrial high-resolution respirometry	*n* = 32 rats	Melatonin enhances mitochondrial function
Halpern et al. (2020)	RCT	Effect of melatonin on BAT	PET-CT	*n* = 19 rats	Melatonin deficiency impairs BAT response
Ramírez-Plascencia et al. (2022)	RCT	Effect of adiponectin and leptin on sleep-wake cycle	Administration of adiponectin or leptin	*n* = 8 rats	Leptin promotes REM and NREM sleep/adiponectin promotes wakefulness
Wei et al. (2022)	Network analysis	Network analysis of 6 adipokines and their molecular partners	Network analysis	Cell culture	Leptin, AMP activated kinase, fatty acid oxidation plays a key role in metabolic and sleep disorders
Choi et al. (2016)	Cell culture	Browning effect of thymol	Real time-PCR	Cell culture	Thymol may have influence in the browning of AT
Kang et al. (2019)	Cell culture	Browning effect of trans-cinnamic acid	Real time-PCR	Cell culture	Trans-cinnamic acid can induce browning of AT
Lee et al. (2016)	RCT	Effect of high fat diet on adipocyte browning	PCR	*n* = 15 mice	Onion peel remodels adipocytes versus brown-like adipocytes
Wirth et al. (2022)	Review	Effect of increased protein intake on sleep outcomes	Database search	*N* = 10 articles	No clear relationship between increased protein intake and sleep
Roshanmehr et al. (2022)	Cross-sectional analysis	Influence of breakfast type on sleep	Online survey	*n* = 2671 (41% women)	Japanese style breakfast is associated with morning preference
van der Merwe et al. (2022)	Review	Does chronotype impact sleeping behavior	Chronotype assessment database search	*n* = 24 articles	Evening chronotypes are more likely to develop obesity
Hermes et al. (2022)	Review	Correlation between nutrition and sleep duration in children	Database search	*n* = 24 articles	Short sleep duration correlates with obesity
Li et al. (2022)	Cross-sectional study	Eating habit patterns and mental health	General health questionnaire	*n* = 1348	Unhealthy eating behavior is correlated with mental health problems
Zhou et al. (2022)	Cross-sectional study	Comparison between health behaviors, depression, and perceived health status between athlete and non-athlete students	Online questionnaire	*n* = 624	Athletes show healthier habits and report lower proportion of depression
Ramón-Arbués et al. (2022)	Cross-sectional study	Association between diet and sleep quality	Pittsburgh Sleep Quality Index	*n* = 868	Unhealthy eaters (more soft drinks and sugar) are more likely to have poor sleep quality
Mattar et al. (2022)	Cross-sectional study	Relationship between ultra-processed foods and sleep	Pathway analysis, questionnaires	*n* = 2826	Short sleep duration is among other factors associated with higher ultra-processed foods consumption
Zarpellon et al. (2022)	Cross-sectional study	Influence of diet on sleep quality in obese children	Sleep disturbance scale for children	*n* = 43	Higher total daily energy intake correlates with poor sleep quality
Duraccio et al. (2022)	Within subjects crossover study	How does short sleep affect dietary consumption	Multiple-pass dietary recall	*n* = 93	Adolescents with insufficient sleep eat unhealthier promoting weight and cardiometabolic outcomes
Doan et al. (2022)	Review	Relationship between sleep durations and eating behaviors	Database search	*n* = 61 articles	Inadequate sleep is associated with binge eating
Arslan et al. (2022)	Cross-sectional study	Effect of chronotype on addictive eating behavior	Morningness-eveningness questionnaire	*N* = 850 (53% women)	Morning types tended to be obese but demonstrated fewer addictive eating behaviors
Rosi et al. (2022)	Cross-sectional study	Relation between chronotype, sleeping, and eating patterns	Morningness-eveningness questionnaire	*n* = 74 (72% women)	Morning types consumed less sweets and ultra-processed food
Champion et al. (2022)	Review	Effect of parent-based interventions on lifestyle risks	Database search	*n* = 46 articles	Partner-based interventions were associated with less lifestyle risks
Webster et al. (2022)	Interview	Monitoring of the management of sleep disturbances in patients with dementia	Structured interview	*n* = 18	Common strategies are insufficient
Spaeth et al. (2020)	RCT	Effect of repetitive sleep restriction on caloric intake	Food monitoring	*n* = 35 (17 women)	Sleep restriction leads to increased caloric intake but it does not cumulatively increase with multiple restriction and is unaffected by recovery opportunities
Arredondo-Amador et al. (2020)	Cross-sectional study	Circadian rhythms in the activity of HLS	Abdominal AT biopsies	*n* = 18	HLS activity depends on the circadian rhythm and is highest around midnight
Seifollahi et al. (2022)	Cross-sectional study	Effect of MIND diet on sleep	Pittsburgh Sleep Quality Index	*n* = 282 women	No significant association between MIND diet and sleep

Abbreviations: *RCT* randomized controlled trial, *AT* adipose tissue, *BAT* brown adipose tissue, *WAT* white adipose tissue, *MIND* Mediterranean-DASH Intervention for Neurodegenerative Delay Diet, *HLS* hormone-sensitive lipase, *PCR* polymerase chain reaction, *PET-CT* positron emission tomography-computed tomography, *REM* rapid eye movement sleep, *NREM* non-rapid eye movement sleep, *AMP* adenosine monophosphate

### Hormonal and neuronal influence of food on sleep and circadian rhythm

According to molecular research, a key for nutritional influence on sleep, sleep quality, and sleep disorders lies in the production of mediators in adipocytes. Both white and brown fat may play a role in circadian rhythm. Contrary to former beliefs that white and brown adipocytes remain constant for a life span, it is now known that white fat may become brown fat or intermediate beige fat and vice versa [16]. Brown adipocytes are the most productive of exogenous agents. The best known agents secreted by adipocytes are bioactive peptides, the adipokines including leptin, resistin, vaspin, visfatin, hepcidin, adiponectin, and inflammatory cytokines [16]. Many neuroactive peptides are also produced. Machado and colleagues reviewed how active brown adipocytes react to the thermogenesis of warming and how thermogenesis and circadian rhythms are dependent upon each other [16]. There is a bidirectional influence on melatonin through uncoupling protein 1 (UCP1) expressed from adipocyte membranes. Melatonin stimulates browning of adipocytes and production of UCP1. UCP1 mRNA then stimulates melatonin expression and thereby stabilizes and re-enforces circadian rhythm and sleep [17]. If there is a disruption of melatonin stimulation for reasons such as insufficient light or reduced sleep, the browning of white fat is disrupted [18]. This may be one explanation on a molecular basis for weight gain associated with insufficient total sleep time.

Other major players in circadian rhythm and sleep are leptin and adiponectin. White fat and brown fat produce both leptin and adiponectin. These metabolic hormones have a direct influence in the clock nuclei involved in the sleep-wake cycle [19, 20]. Leptin and adiponectin influence the ventrolateral preoptic nuclei (VLPO), a major area for sleep promotion [19]. Adiponectin administration increases wakefulness during the rest phase, reduces delta power, and activates wake-promoting neurons, such as those in the locus coeruleus (LC), tuberomammillary nucleus (TMN), and hypocretin/orexin neurons (OX) within the lateral hypothalamus (LH) and perifornical area (PeF). Conversely, leptin promotes REM and NREM sleep, including an increase of delta power during NREM sleep. Leptin induces c-Fos expression in VLPO and melanin concentrating hormone-expressing neurons (MCH) [19].

Most of this neuronal and mediator research has been performed in animals. It remains to be seen if these findings can be substantiated in human physiology.

### Influence of certain foods on adipocyte mediator expression and on adipocyte browning or whitening

There are few nutritional substances that have a direct influence on brown adipocytes in animals and stimulate the production of UCP1. Curcumin is found in the spices saffron, thyme, and cinnamon as well as in quercetin contained in onions. Other substances such as pepper and capsaicin may work only in combination in the presence of other mediators. Research on these spices and chemicals is restricted to knock out mice [21–23]. The application of these findings to humans remains speculative in view of the large doses of chemicals used in this animal research.

### No direct effect of certain food substances on sleep

The search for studies on the effect of particular nutritional substances on sleep revealed only two serious publications. The first is a meta-analysis of studies investigating effects of increased protein intake on circadian rhythm and sleep. This study concluded that there is no real effect of increased protein intake on sleep [24]. The only effect in the majority of studies is a subjective improvement of sleep quality demonstrated subjectively by questionnaires.

In a survey of over 2000 people, subjects were asked about their type of breakfast (Japanese style with rice, algae, and proteins, Western style with mainly bread or mainly cereal, and alternating Western and Japanese style) and chronotype (morning person vs evening person). Results showed that a Japanese style breakfast is strongly associated with a morning active chronotype and the other breakfast styles are more associated with evening chronotypes [25]. However, these results are based on subjective answers and one may speculate that persons with a so-called healthy diet and lifestyle consider themselves as morning type compared to people with a less healthy diet and who describe themselves as active evening chronotypes. A direct influence on sleep or sleep-wake cycle by food cannot be concluded from these two reports.

A very recent meta-analysis of articles on food intake analyzed 24 studies on this topic [26]. The meta-analysis concluded that morning chronotype has a more healthy eating behavior than the evening type who eats more fat and carbohydrate-containing food than the morning chronotype. All the analyzed studies were based on subjective questionnaires. There have been no data derived from objective measurement of sleep or activity via actigraphy.

### Children and adolescent eating behaviors, late sleep onset, and short sleep

There is a larger number of publications that correlate nutrition in children, adolescents, and sleep parameters. These studies present fairly uniform conclusions.

A review of 24 articles of sleep and nutrition in children revealed that a majority of original studies associated missing nutrients and a high amount of sugar in the diets of obese children with insufficient total sleep time. A minority of studies presented a link between obesity and food with sleep parameters [27].

There has been an increase in the number of studies on lifestyle and sleep behavior in adolescents recently. Late consumption of carbohydrates was associated with shorter sleep in Chinese adolescents though this association was complicated by sleep restriction linked to depression and other mental health problems in young people [28]. However, young Chinese athletes with a healthy lifestyle found that a healthier diet gave them the best results in competition. The sleep of these athletes was less fragmented and more stable with an overall higher sleep quality and less signs of depressive mood [29]. A problem with these two studies is the unclear use of questionnaires and the lack of objective data collection (e.g., polysomnography, polygraphy, actigraphy) and the possible bias towards a push for a so-called healthy diet.

In a sample of over 800 students, data collection was based on validated questionnaires such as the Pittsburg Sleep Quality Index (PSQI) [30]. There was a high odds ratio for low sleep quality in the so-called unhealthy eaters (i.e., those who ate more sweets and meat and less vegetables etc.). However, a majority of young women volunteered for the survey (67%) and they considered themselves to be healthy eaters, leaving a small minority of unhealthy eaters as the focus of the study. There were a number of issues that biased the outcome of the investigation.

A large number of investigations [31–33] were based upon epidemiologic studies in children, adolescents, and young adults concerning sleep duration, time online. or time on computer gaming and the consumption of fast food or ultra-processed foods. A systematic review listed a total of 61 such studies [34]. Almost all studies concluded that there is a correlation of short sleep due to working late, gaming into the night, increased consumption of ultra-processed food, and a risk for obesity. However, none of these investigations showed direct disruption of sleep or shortening of sleep by intake of ultra-processed food. A direct connection between length of sleep and particular food intake has not been established.

One study enrolled 850 students equally divided between the genders and showed that morning chronotype tended to increase BMI and change eating behavior [35]. However, the study clearly showed that the questionnaire survey results for sleep, chronotype, and eating behavior differ at different time points in adolescents or young adults.

In another study [36], eating behavior and late sleep onset time do not differ much between adolescents and adults.

The effect of parental education on changing consumption in teenagers is moderate according to a recent review of randomized controlled studies on lifestyle behavioral change [37]. Parents were able to achieve a slight increase in physical exercise, shortened screen time, earlier bedtime with longer sleep time, and reduced junk food consumption.

Changing sleep patterns in nursing home residents with dementia via food intake was described in a survey of nursing staff [38]. Nursing staff often encourages demented residents to eat sufficiently during day time and have only a snack for dinner in the belief that this keeps the residents asleep at night. If demented residents nevertheless have a chaotic sleep-wake cycle and get up at night, the nursing staff keeps them awake with coffee or tea in addition to activities in order to get them tired. While this seems a practical approach in elderly demented patients, the authors concluded that the caffeine delivery negatively influences the sleep-wake cycle. There has been no controlled trial to determine if certain food, certain amounts of food, or certain meal times may help persons with dementia to sleep at night.

### Fat metabolism and sleep

In a randomized controlled trial [39] with 224 participants, 198 subjects were sleep restricted to 4 h time in bed (TIB) and 27 controls subjects received 10 h TIB over a period of 5 nights. With a control group of 10 subjects, 35 participants spent after two baseline nights ten nights with restricted sleep (4 h) in two exposures of five nights each. Daily calorie intake increased by an impressive 527 kcal per day in the sleep-restricted subjects versus control subjects and sleep-restricted subjects gained an average of 0.86 kg over the period. The study results strongly suggested a connection between sleep restriction and weight gain. A possible mechanism of this effect of sleep restriction may be the influence of the sleep-wake cycle on hormones affecting adipocytes.

Another study approached the issue of timing of food intake [40] by measuring the influence of circadian rhythm on hormone-sensitive lipase, a fat dissolving enzyme. The results showed that the later that dinner was eaten after 10:00 pm, the lower the effect of lipase. With lipase most active around midnight, longer duration of fasting at night was associated with higher fat mobilization and fat burning. The findings suggest that the sleep-wake cycle affects the role of fat dissolving enzymes on metabolism.

Unfortunately there are no human studies that investigate how nutrition may influence sleep. A large trial in mildly obese women who were followed for several weeks found no influence of the MIND diet (Mediterranean-DASH Intervention for Neurodegenerative Delay Diet) on sleep quality assessed with the Pittsburgh Sleep Quality Index questionnaire [41]. The MIND diet combined two forms of so-called healthy nutrition with low amounts of both carbohydrates and saturated fat and increased amounts of unsaturated fat and vegetables.

Other weight loss programs and diets have been unable to show any influence on sleep parameters [42–48] though there may be gender differences concerning success with weight loss and the effects of behavioral changes on bedtimes and nutrition [43].

## Conclusions

The question if certain food or nutrition can influence sleep parameters and sleep quality remains unanswered. Based upon the existing literature, there is no evidence that certain types of food have a measurable effect on sleep, either subjectively as assessed by validated questionnaires or objectively when measured via polysomnography or wearable devices.

There is evidence that short or fragmented sleep, late onset sleep with late dinners, and late chronotypes do have an influence on metabolism. A possible mechanism for these effects may be mediated by adipocyte-derived hormones.

In summary, the effects of food and nutrition on the human body, including sleep, are probably overestimated because of the influence of autoregulation mechanisms [49].

## Data Availability

Data will be made available on reasonable request.
